# Supplementary material for: Construction of an arrayed CRISPRi library as a resource for essential gene function studies in Streptococcus mutans
Source: Microbiol Spectr. 2023 Dec 6;12(1):e03149-23. doi: 10.1128/spectrum.03149-23 (PMC10783072; doi:10.1128/spectrum.03149-23)
Supplement: Supplemental material — Fig. S1 to S4 and Table S1. [file spectrum.03149-23-s0001.docx]

**Supplemental Material for:**

**Title: Construction of an arrayed CRISPRi library as a resource for essential gene function studies in *Streptococcus mutans***

**Authors**: Jackson St. Pierre^1,2^, Justin Roberts^3,4^, Mohammad A. Alam^3^, Robert C. Shields^1*^

^1^Department of Biological Sciences, Arkansas State University, Jonesboro, Arkansas

^2^New York Institute of Technology College of Osteopathic Medicine, Jonesboro, Arkansas

^3^Department of Chemistry & Physics, Arkansas State University, Jonesboro, Arkansas

^4^University of Arkansas for Medical Sciences, Little Rock, Arkansas

**This file includes:**

Figs. S1 – S4

Table S1


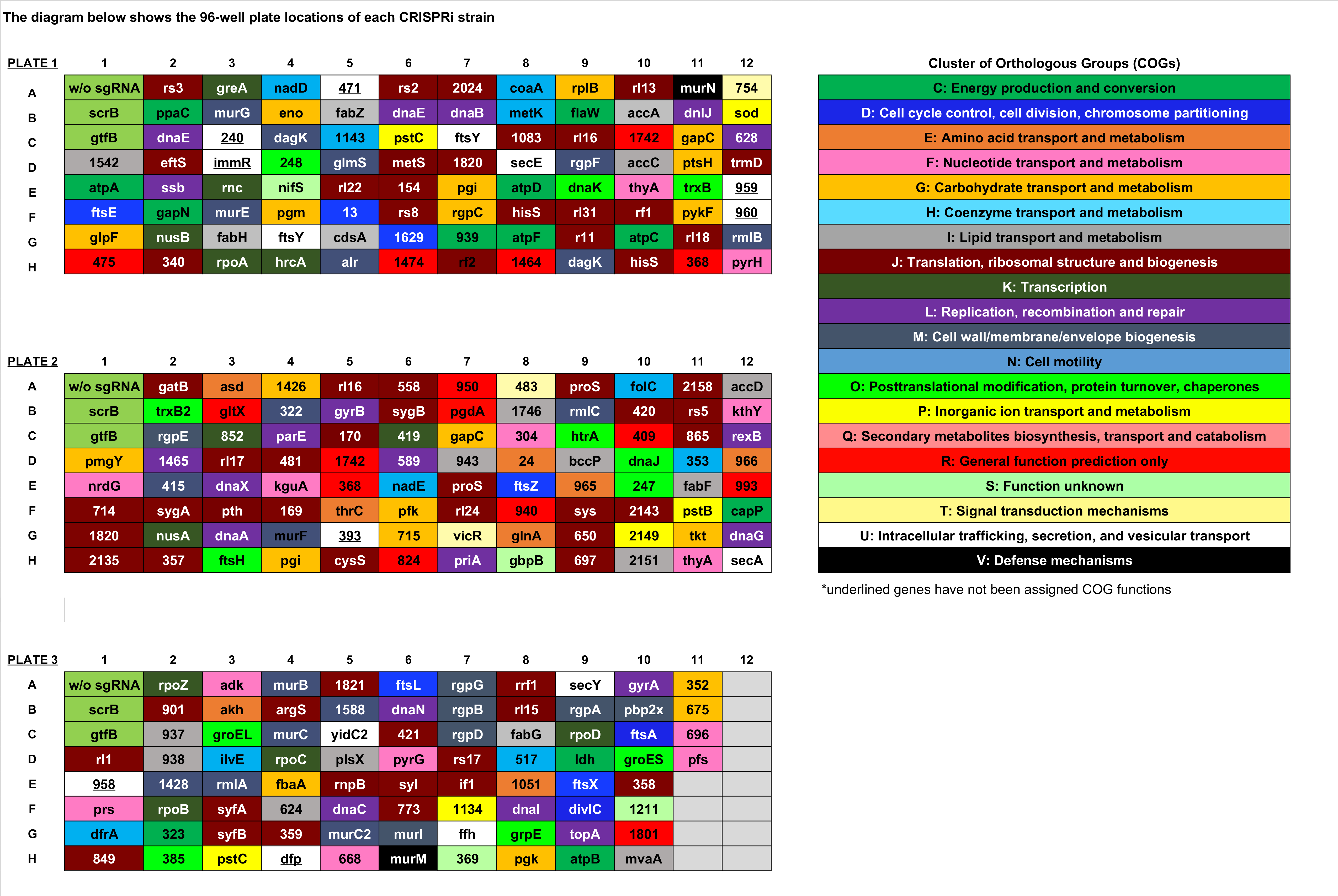


**Figure S1 SNAP library plate layout.** This diagram shows the location of each CRISPRi strain within the three SNAP plates. Each of the three plates has control strains in A1, B1, and C1. Plate 3 has a total of 84 CRISPRi strains. Each CRISPRi strain is also color coded according to the putative functional pathway that the sgRNA targets (COG; Cluster of Orthologous Groups).


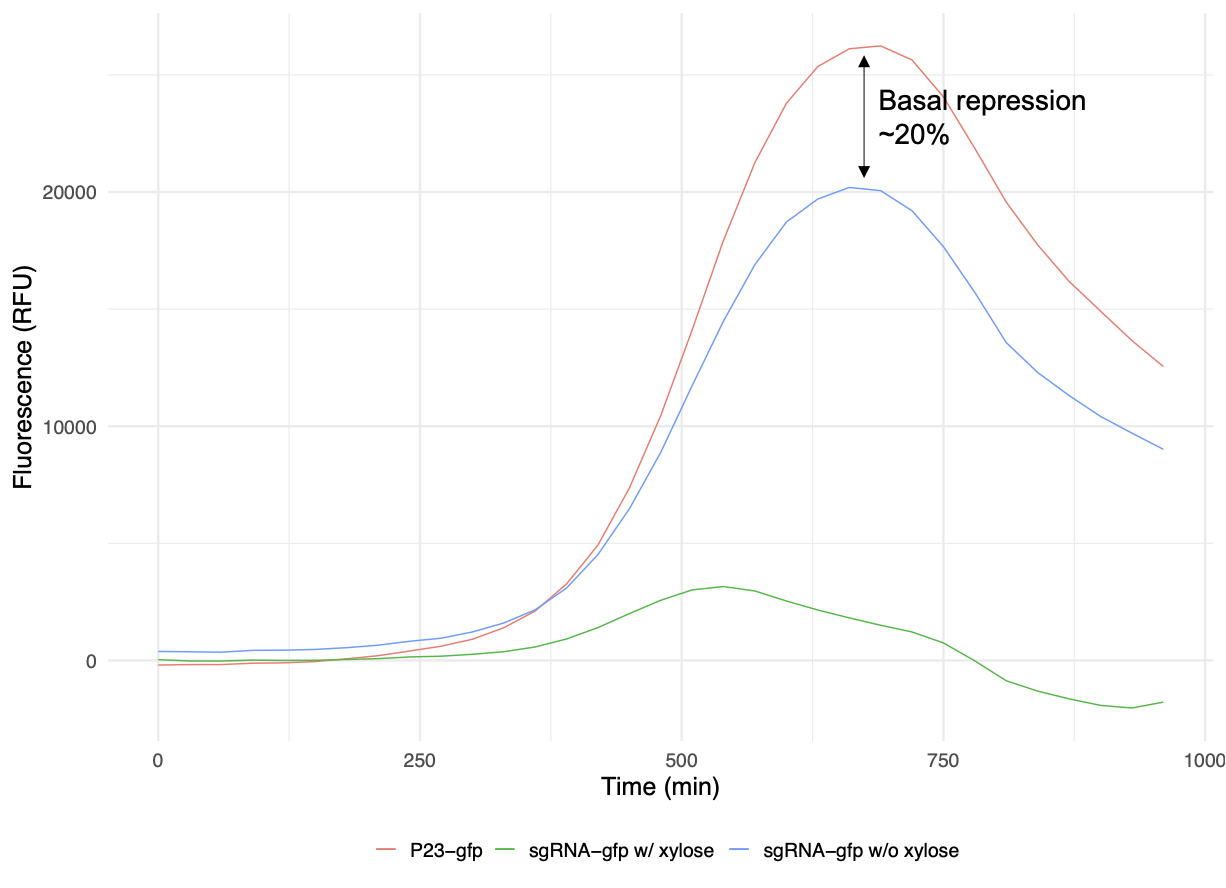


**Figure S2 sgRNA*^gfp^* leads to basal repression of GFP production.** To investigate whether P*_xyl_* basal activity leads to repression of target gene expression without xylose addition, we compared GFP production in *S. mutans* Δcas9 P*_xyl_*-dcas9 and the same strain also carrying sgRNA*^gfp^*. There was a notable ~20% decline in GFP production in the strain targeting *gfp* but without the addition of xylose. With 0.1% xylose added, repression of GFP production was ~95%.


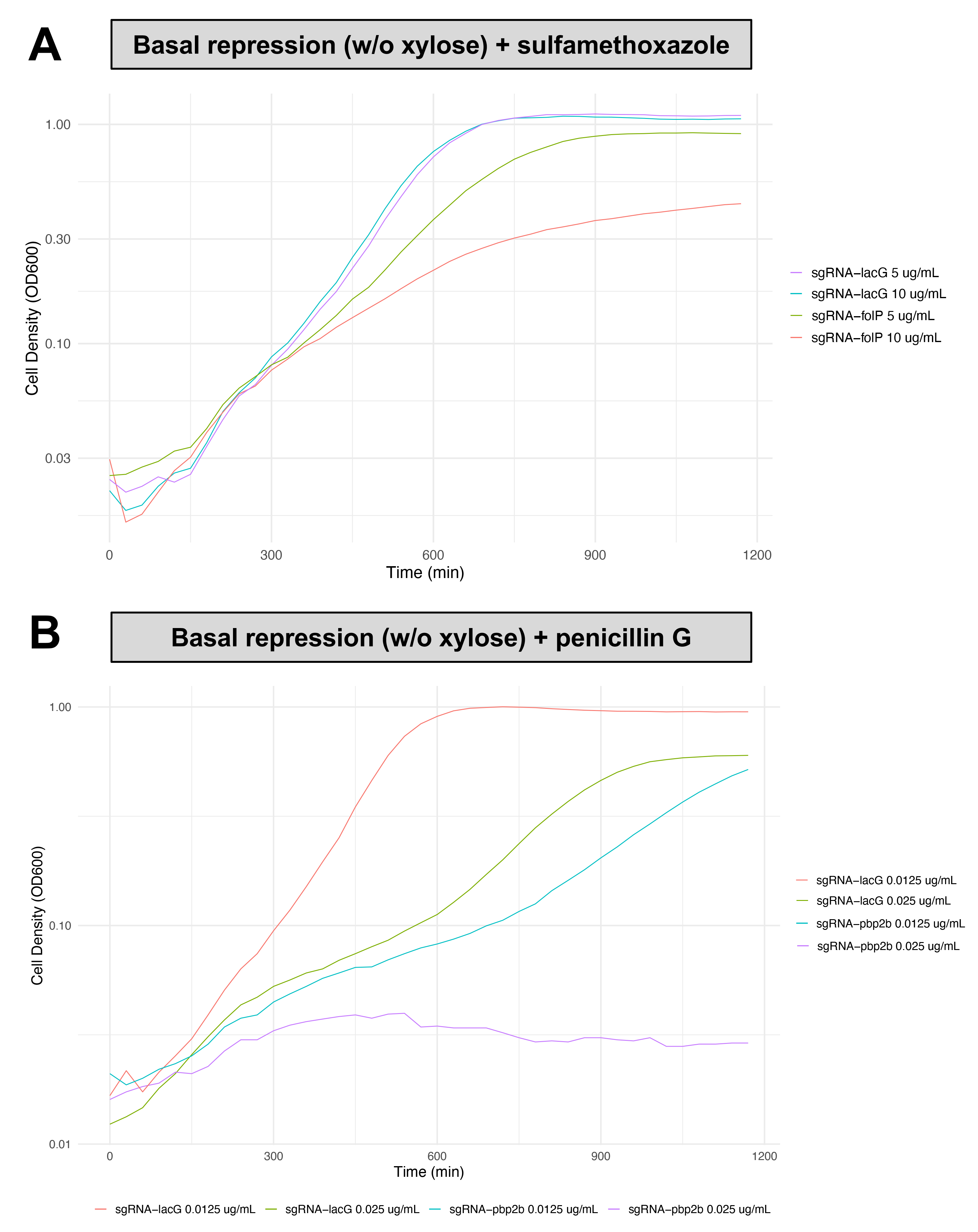


**Figure S3 Basal repression of genes by CRISPRi sensitizes *S. mutans* to antimicrobials.** sgRNA plasmids were constructed that target *folP* (dihydropteroate synthase) and *pbp2b* (penicillin-binding protein 2b). When compared to a control strain targeting *lacG*, sgRNA*^folP^* was more sensitive to sulfamethoxazole (SMX), an antibiotic that interferes with folic acid synthesis (A). Targeting pbp2b increased the sensitivity of *S. mutans* to penicillin G (PenG; benzylpenicillin), a β-lactam antibiotic that targets cell wall synthesis by binding to penicillin binding proteins (B). Growth curves are representative of three biological replicates.


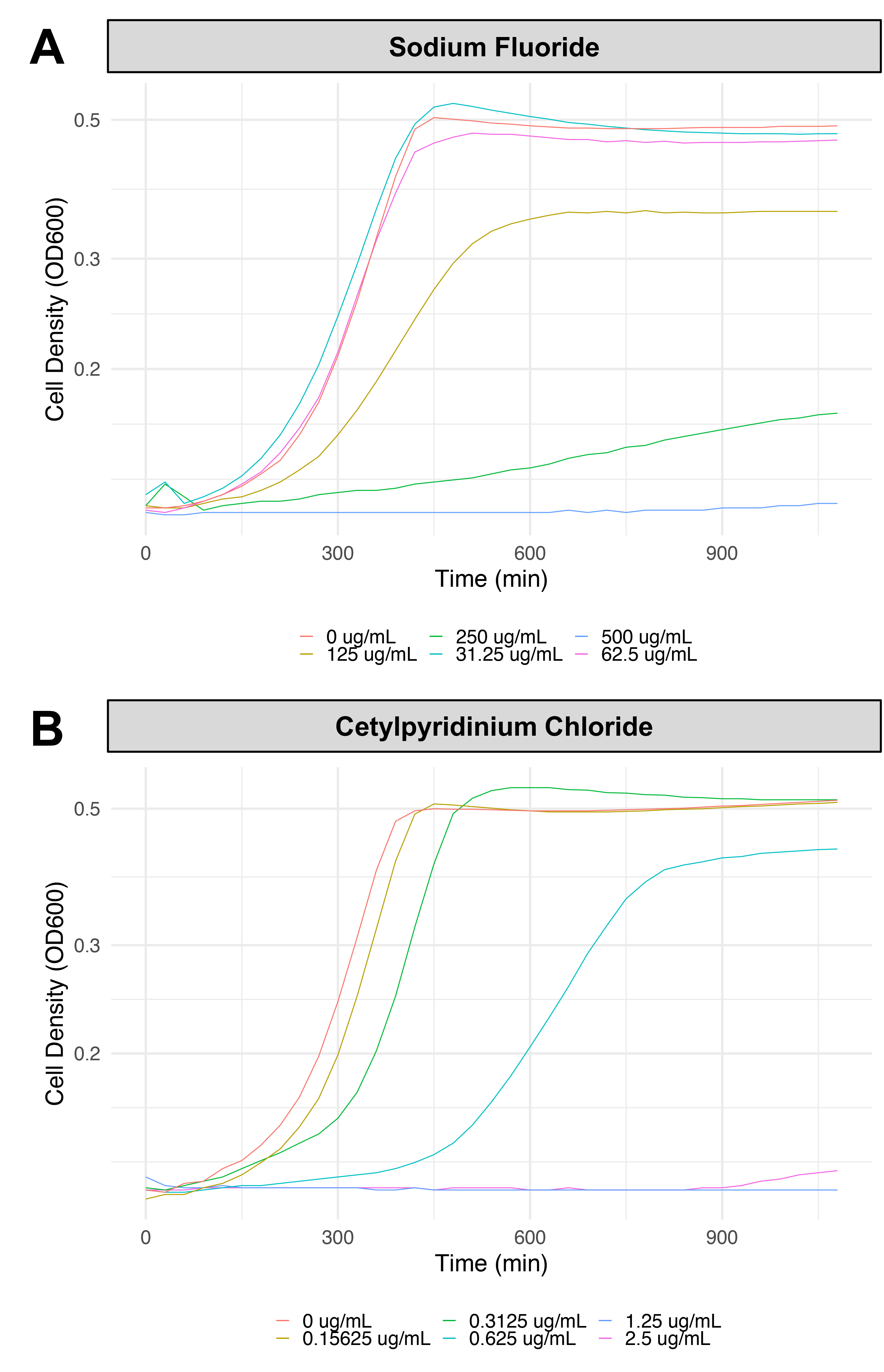


**Figure S4 Minimum inhibitory concentrations of sodium fluoride (NaF) and cetylpyridinium chloride (CPC) against *S. mutans*.** A broth dilution method was used to measure the minimum inhibitory concentration (MIC) of NaF (A) and CPC (B). Bacterial growth was monitored using a Bioscreen C Pro automated system. Growth curves are representative of three biological replicates.

**Table S1 AUC_sgRNA_/AUC_control_ raw data for BHI, CDM-Xylose, NaF and CPC microplate growth experiments.**

|  | **Condition** | | | | | | | | | | | |
| --- | --- | --- | --- | --- | --- | --- | --- | --- | --- | --- | --- | --- |
| **sgRNA target** | **BHI-1** | **BHI-2** | **BHI-3** | **CDM Xylose-1** | **CDM Xylose-2** | **CDM Xylose-3** | **NaF-1** | **NaF-2** | **NaF-3** | **CPC-1** | **CPC-2** | **CPC-3** |
| **rs3** | 0.72 | 1.17 | 1.48 | 0.14 | 0.09 | 0.03 | 1.31 | 0.62 | 0.28 | 0.92 | 0.97 | 0.85 |
| **greA** | 1.00 | 1.07 | 1.35 | 0.73 | 1.25 | 0.87 | 1.31 | 0.67 | 0.83 | 0.53 | 0.86 | 0.89 |
| **nadD** | 1.01 | 0.88 | 1.16 | 0.86 | 1.19 | 0.70 | 1.58 | 0.75 | 1.02 | 0.84 | 0.99 | 1.01 |
| **471** | 0.79 | 0.95 | 1.43 | 0.32 | 0.07 | 0.00 | 1.54 | 0.56 | 1.06 | 0.80 | 1.14 | 0.93 |
| **rs2** | 1.01 | 1.18 | 1.27 | 0.21 | 0.03 | 0.03 | 1.16 | 0.60 | 0.92 | 1.03 | 0.88 | 1.08 |
| **2024** | 0.95 | 1.04 | 1.38 | 0.19 | 0.02 | 0.02 | 1.66 | 1.03 | 0.81 | 0.59 | 1.20 | 0.83 |
| **coaA** | 0.07 | 1.12 | 1.35 | 0.03 | 0.02 | 0.00 | 1.13 | 0.90 | 1.07 | 0.03 | 1.03 | 2.03 |
| **rplB** | 0.84 | 1.11 | 1.24 | 0.50 | 1.10 | 1.07 | 1.46 | 1.17 | 1.01 | 0.64 | 0.97 | 0.91 |
| **r13** | 0.81 | 1.18 | 1.12 | 0.34 | 0.74 | 0.44 | 1.36 | 0.94 | 0.84 | 0.59 | 1.00 | 0.77 |
| **murN** | 0.60 | 1.00 | 0.79 | 0.44 | 0.25 | 0.08 | 1.32 | 0.76 | 0.91 | 0.77 | 1.04 | 0.80 |
| **754** | 0.54 | 1.13 | 0.83 | 0.55 | 1.15 | 0.28 | 1.21 | 0.62 | 1.21 | 0.57 | 0.78 | 0.87 |
| **ppaC** | 0.70 | 1.12 | 1.55 | 0.33 | 0.77 | 0.49 | 1.38 | 0.92 | 0.69 | 0.61 | 0.73 | 1.11 |
| **murG** | 1.07 | 1.12 | 1.34 | 0.25 | 0.04 | 0.02 | 1.14 | 0.98 | 0.82 | 0.69 | 0.76 | 1.02 |
| **eno** | 0.89 | 1.00 | 1.17 | 0.30 | 0.06 | 0.03 | 1.14 | 0.76 | 0.90 | 0.90 | 0.77 | 0.94 |
| **fabZ** | 0.55 | 0.70 | 0.87 | 0.20 | 0.04 | 0.03 | 1.19 | 0.63 | 0.96 | 0.52 | 0.61 | 0.52 |
| **dnaE** | 0.89 | 1.01 | 1.16 | 0.59 | 0.85 | 0.62 | 1.17 | 0.96 | 0.85 | 0.13 | 0.11 | 0.66 |
| **dnaB** | 0.83 | 1.17 | 1.12 | 0.51 | 0.16 | 0.08 | 1.48 | 0.68 | 0.87 | 0.45 | 1.19 | 0.80 |
| **metK** | 0.82 | 0.87 | 1.36 | 0.47 | 0.63 | 0.21 | 1.57 | 0.56 | 1.23 | 0.54 | 0.85 | 1.01 |
| **flaW** | 0.81 | 1.20 | 1.33 | 0.70 | 0.99 | 0.84 | 1.21 | 0.26 | 1.08 | 0.61 | 1.00 | 0.75 |
| **accA** | 1.00 | 1.01 | 1.10 | 0.26 | 0.14 | 0.13 | 1.13 | 0.84 | 1.25 | 0.53 | 0.56 | 0.54 |
| **dnlJ** | 0.65 | 1.11 | 1.44 | 0.32 | 0.30 | 0.08 | 1.54 | 1.04 | 0.76 | 0.65 | 0.80 | 0.68 |
| **sod** | 1.04 | 1.15 | 1.20 | 0.95 | 0.97 | 0.85 | 1.49 | 1.27 | 0.78 | 0.51 | 0.93 | 0.74 |
| **dnaE** | 0.65 | 1.08 | 1.20 | 0.25 | 0.74 | 0.73 | 1.32 | 0.87 | 1.16 | 0.53 | 0.61 | 1.29 |
| **240** | 0.82 | 1.08 | 1.24 | 0.53 | 0.56 | 0.38 | 1.15 | 0.43 | 0.88 | 0.73 | 1.02 | 0.76 |
| **dagK** | 0.89 | 1.04 | 1.28 | 0.62 | 1.02 | 0.76 | 1.56 | 0.91 | 0.84 | 0.71 | 0.81 | 1.02 |
| **1143** | 1.14 | 0.87 | 1.08 | 0.88 | 0.85 | 0.85 | 1.25 | 0.66 | 0.89 | 0.48 | 0.68 | 0.82 |
| **pstC** | 0.84 | 0.85 | 1.10 | 0.24 | 0.76 | 0.62 | 1.05 | 0.79 | 0.91 | 0.78 | 0.99 | 0.96 |
| **ftsY** | 0.92 | 0.92 | 1.29 | 0.53 | 1.12 | 1.17 | 1.66 | 0.94 | 0.90 | 0.69 | 1.21 | 0.82 |
| **1083** | 0.70 | 0.94 | 1.32 | 0.42 | 0.46 | 0.11 | 1.37 | 0.48 | 0.93 | 0.42 | 0.72 | 0.69 |
| **rl16** | 0.67 | 0.89 | 1.11 | 0.17 | 0.20 | 0.05 | 0.81 | 0.19 | 0.71 | 0.54 | 0.68 | 0.73 |
| **1742** | 0.76 | 0.83 | 1.24 | 0.13 | 0.02 | 0.01 | 1.19 | 1.15 | 0.84 | 0.40 | 0.50 | 0.48 |
| **gapC** | 0.55 | 1.00 | 1.42 | 0.14 | 0.08 | 0.06 | 1.45 | 0.50 | 0.79 | 0.62 | 0.50 | 0.81 |
| **628** | 0.82 | 0.84 | 1.37 | 1.13 | 1.17 | 0.83 | 1.62 | 0.26 | 0.75 | 0.66 | 0.40 | 1.02 |
| **1542** | 0.80 | 0.96 | 1.20 | 0.41 | 0.10 | 0.04 | 1.20 | 1.41 | 0.94 | 0.80 | 1.02 | 0.99 |
| **eftS** | 0.76 | 0.97 | 1.11 | 0.20 | 0.16 | 0.07 | 1.29 | 0.83 | 0.86 | 0.83 | 0.98 | 0.94 |
| **218** | 0.85 | 1.17 | 1.41 | 0.47 | 0.26 | 0.04 | 1.44 | 0.81 | 0.87 | 0.28 | 0.29 | 0.61 |
| **248** | 0.76 | 0.97 | 1.18 | 0.80 | 1.03 | 0.84 | 1.67 | 1.38 | 0.95 | 0.78 | 0.67 | 0.87 |
| **glmS** | 0.85 | 0.91 | 1.18 | 0.57 | 0.90 | 1.07 | 1.26 | 1.16 | 0.82 | 0.73 | 0.80 | 0.70 |
| **metS** | 0.90 | 0.95 | 1.40 | 0.19 | 0.10 | 0.04 | 1.38 | 0.99 | 0.92 | 0.44 | 0.49 | 0.85 |
| **1820** | 0.81 | 1.04 | 1.44 | 0.54 | 1.27 | 1.16 | 1.20 | 0.85 | 0.82 | 0.61 | 1.25 | 0.93 |
| **secE** | 0.59 | 0.91 | 1.39 | 0.42 | 0.85 | 0.39 | 1.35 | 0.70 | 0.73 | 0.79 | 1.15 | 0.93 |
| **rgpF** | 0.98 | 1.09 | 1.32 | 0.52 | 0.57 | 0.23 | 1.40 | 1.01 | 0.81 | 1.07 | 1.09 | 1.04 |
| **accC** | 0.78 | 0.96 | 1.31 | 0.18 | 0.04 | 0.03 | 1.33 | 0.89 | 1.02 | 0.31 | 0.57 | 0.56 |
| **ptsH** | 0.66 | 0.99 | 1.24 | 0.28 | 0.21 | 0.15 | 1.40 | 0.47 | 0.86 | 0.58 | 0.79 | 1.00 |
| **trmD** | 0.88 | 0.84 | 1.41 | 1.56 | 0.92 | 0.53 | 1.64 | 0.50 | 0.74 | 0.86 | 0.87 | 0.69 |
| **atpA** | 0.60 | 1.01 | 1.23 | 0.08 | 0.49 | 0.53 | 1.43 | 1.16 | 1.14 | 0.76 | 0.98 | 0.77 |
| **ssb** | 0.68 | 0.89 | 1.11 | 0.14 | 0.35 | 0.20 | 1.24 | 0.91 | 0.56 | 0.32 | 0.29 | 0.84 |
| **rnc** | 1.28 | 1.07 | 1.33 | 0.69 | 0.98 | 0.63 | 1.15 | 1.33 | 0.43 | 0.20 | 0.40 | 0.55 |
| **nifS** | 0.89 | 0.95 | 1.13 | 0.83 | 1.15 | 1.08 | 1.25 | 0.80 | 0.75 | 0.76 | 0.72 | 0.88 |
| **rl22** | 0.99 | 0.72 | 0.86 | 0.30 | 0.32 | 0.04 | 1.34 | 0.65 | 0.68 | 0.34 | 0.60 | 0.64 |
| **154** | 0.94 | 1.17 | 1.34 | 0.25 | 0.70 | 0.32 | 1.27 | 1.21 | 1.11 | 0.88 | 0.97 | 1.00 |
| **pgi** | 0.73 | 1.00 | 1.33 | 0.26 | 0.24 | 0.13 | 1.15 | 0.91 | 0.84 | 0.44 | 0.84 | 0.69 |
| **atpD** | 0.61 | 1.03 | 1.31 | 0.17 | 0.50 | 0.45 | 1.31 | 0.50 | 0.99 | 0.83 | 1.22 | 0.98 |
| **dnaK** | 0.93 | 1.03 | 1.28 | 0.39 | 0.54 | 0.37 | 1.69 | 0.30 | 1.07 | 0.71 | 0.85 | 0.89 |
| **thyA** | 0.65 | 1.09 | 1.58 | 0.27 | 0.62 | 0.63 | 1.21 | 0.74 | 0.84 | 0.50 | 0.63 | 0.77 |
| **trxB** | 0.65 | 1.08 | 1.41 | 0.37 | 1.03 | 0.99 | 1.52 | 0.64 | 0.93 | 0.78 | 1.05 | 1.05 |
| **959** | 0.79 | 1.13 | 1.49 | 0.94 | 1.12 | 0.81 | 1.39 | 0.93 | 0.75 | 0.59 | 0.60 | 0.83 |
| **ftsE** | 0.77 | 0.91 | 1.19 | 0.43 | 0.54 | 0.09 | 1.43 | 1.06 | 0.98 | 0.77 | 0.96 | 1.02 |
| **gapN** | 0.85 | 1.07 | 1.27 | 0.22 | 0.08 | 0.13 | 1.18 | 1.00 | 0.93 | 0.45 | 0.22 | 0.65 |
| **murE** | 1.02 | 1.03 | 1.14 | 0.36 | 0.06 | 0.03 | 1.11 | 1.04 | 0.79 | 0.09 | 0.17 | 0.10 |
| **pgm** | 0.89 | 1.01 | 1.23 | 0.44 | 0.33 | 0.16 | 1.44 | 1.59 | 0.92 | 0.18 | 0.57 | 0.36 |
| **13** | 0.96 | 1.10 | 1.22 | 0.60 | 0.96 | 0.74 | 1.30 | 1.19 | 0.84 | 0.64 | 1.13 | 0.90 |
| **rs8** | 0.92 | 1.19 | 1.49 | 0.21 | 0.06 | 0.02 | 1.38 | 0.89 | 0.96 | 0.91 | 0.85 | 0.87 |
| **rgpC** | 0.77 | 1.10 | 1.27 | 0.29 | 0.39 | 0.17 | 1.30 | 0.88 | 0.76 | 0.68 | 1.11 | 0.91 |
| **hisS** | 0.70 | 0.97 | 1.60 | 0.35 | 0.17 | 0.12 | 1.62 | 0.65 | 0.99 | 0.79 | 1.22 | 1.10 |
| **rl31** | 0.82 | 1.18 | 1.29 | 0.39 | 0.65 | 0.44 | 1.48 | 0.46 | 1.02 | 0.57 | 0.52 | 0.81 |
| **rf1** | 0.69 | 1.03 | 1.40 | 0.39 | 1.03 | 0.77 | 1.33 | 0.95 | 0.65 | 0.76 | 0.97 | 0.93 |
| **pykF** | 0.64 | 0.98 | 1.07 | 0.60 | 0.96 | 0.89 | 1.49 | 1.06 | 1.00 | 0.60 | 0.95 | 0.77 |
| **960** | 0.60 | 1.15 | 1.55 | 0.22 | 0.10 | 0.03 | 1.40 | 1.07 | 0.93 | 0.67 | 0.91 | 0.91 |
| **glpF** | 0.64 | 0.88 | 1.00 | 0.37 | 1.18 | 1.00 | 1.43 | 0.99 | 0.96 | 0.76 | 0.82 | 0.87 |
| **nusB** | 0.79 | 1.00 | 1.23 | 0.45 | 0.97 | 1.07 | 1.41 | 1.05 | 1.22 | 0.87 | 0.87 | 1.17 |
| **fabH** | 0.86 | 0.91 | 1.15 | 0.29 | 0.07 | 0.08 | 1.20 | 0.71 | 0.91 | 0.69 | 0.98 | 0.95 |
| **ftsY** | 0.72 | 0.88 | 1.15 | 0.59 | 1.11 | 0.85 | 1.39 | 0.97 | 0.86 | 0.87 | 1.18 | 0.90 |
| **cdsA** | 0.97 | 1.02 | 1.31 | 0.28 | 0.04 | 0.04 | 1.24 | 1.08 | 0.97 | 0.50 | 0.32 | 0.97 |
| **1629** | 0.79 | 1.14 | 1.49 | 0.51 | 1.14 | 0.99 | 1.24 | 1.15 | 1.08 | 0.95 | 1.02 | 1.04 |
| **939** | 0.82 | 1.01 | 1.18 | 0.40 | 0.69 | 0.20 | 1.22 | 1.25 | 0.90 | 0.98 | 1.35 | 1.13 |
| **atpF** | 0.85 | 1.00 | 1.32 | 0.18 | 0.41 | 0.12 | 1.24 | 0.91 | 0.93 | 0.69 | 1.25 | 1.02 |
| **r11** | 0.82 | 1.08 | 1.02 | 0.26 | 0.05 | 0.03 | 1.28 | 0.90 | 0.83 | 1.02 | 1.18 | 1.18 |
| **atpC** | 0.74 | 1.05 | 1.19 | 0.13 | 0.57 | 0.54 | 1.33 | 0.62 | 0.73 | 0.48 | 0.85 | 0.70 |
| **rl18** | 0.65 | 1.14 | 1.28 | 0.13 | 0.03 | 0.02 | 1.44 | 1.13 | 0.76 | 0.36 | 0.86 | 0.76 |
| **rmlB** | 1.10 | 0.98 | 1.33 | 1.27 | 0.22 | 0.06 | 1.30 | 1.19 | 0.99 | 0.70 | 0.67 | 0.75 |
| **475** | 0.60 | 0.96 | 1.15 | 0.28 | 0.94 | 0.63 | 1.52 | 1.19 | 0.94 | 0.74 | 0.82 | 0.84 |
| **340** | 0.71 | 1.11 | 1.30 | 0.35 | 0.71 | 0.43 | 1.29 | 0.53 | 0.90 | 0.74 | 0.96 | 1.02 |
| **rpoA** | 0.82 | 0.96 | 1.26 | 0.19 | 0.04 | 0.02 | 1.63 | 0.15 | 0.96 | 0.84 | 1.19 | 0.92 |
| **hrcA** | 0.70 | 0.88 | 1.14 | 0.42 | 0.99 | 0.98 | 1.33 | 1.73 | 0.90 | 0.58 | 0.93 | 0.61 |
| **alr** | 0.67 | 0.95 | 1.50 | 0.37 | 1.00 | 0.99 | 1.24 | 1.16 | 0.97 | 0.87 | 1.11 | 0.88 |
| **1474** | 0.72 | 1.23 | 1.24 | 0.30 | 1.30 | 1.12 | 1.31 | 1.05 | 1.06 | 0.82 | 0.89 | 0.93 |
| **rf2** | 0.70 | 1.26 | 1.55 | 0.39 | 1.15 | 1.14 | 1.31 | 1.07 | 0.97 | 0.75 | 1.37 | 1.08 |
| **1464** | 0.65 | 1.22 | 1.27 | 0.35 | 0.66 | 0.45 | 1.49 | 1.04 | 1.02 | 0.94 | 1.19 | 1.15 |
| **dagK** | 0.70 | 1.06 | 1.67 | 0.35 | 1.02 | 1.06 | 1.83 | 1.14 | 1.04 | 0.72 | 1.18 | 1.13 |
| **hisS** | 0.66 | 1.10 | 1.52 | 0.32 | 0.32 | 0.22 | 1.67 | 1.21 | 0.54 | 0.29 | 1.10 | 0.93 |
| **368** | 1.12 | 1.11 | 1.31 | 1.03 | 0.84 | 0.36 | 1.46 | 1.00 | 0.37 | 0.12 | 0.48 | 0.54 |
| **pyrH** | 1.07 | 1.12 | 1.14 | 1.30 | 0.04 | 0.02 | 1.15 | 0.93 | 0.79 | 0.17 | 1.05 | 1.01 |
| **gatB** | 0.98 | 0.95 | 0.61 | 0.40 | 0.10 | 0.09 | 0.95 | 0.33 | 0.88 | 0.81 | 1.23 | 1.27 |
| **asd** | 0.84 | 1.18 | 0.61 | 0.69 | 1.12 | 1.32 | 0.87 | 0.58 | 0.77 | 0.89 | 1.43 | 1.40 |
| **1426** | 0.96 | 1.00 | 0.79 | 0.66 | 1.05 | 1.38 | 0.59 | 0.39 | 0.99 | 0.84 | 1.61 | 1.16 |
| **r16** | 0.72 | 1.07 | 0.52 | 0.12 | 0.09 | 0.06 | 0.43 | 0.57 | 0.70 | 0.73 | 1.21 | 1.15 |
| **558** | 0.81 | 1.15 | 0.93 | 0.26 | 0.11 | 0.13 | 1.04 | 0.74 | 1.24 | 1.21 | 1.36 | 1.51 |
| **950** | 0.78 | 1.05 | 0.73 | 0.65 | 1.18 | 1.30 | 0.96 | 0.64 | 1.26 | 1.09 | 1.60 | 1.45 |
| **483** | 0.98 | 0.96 | 1.06 | 0.71 | 1.19 | 1.56 | 0.77 | 0.66 | 1.01 | 1.02 | 1.44 | 0.99 |
| **proS** | 0.75 | 1.03 | 0.67 | 0.46 | 0.22 | 0.24 | 1.15 | 0.55 | 0.73 | 1.04 | 1.33 | 1.18 |
| **folC** | 0.94 | 1.24 | 0.71 | 1.03 | 1.18 | 1.48 | 0.73 | 0.51 | 1.13 | 0.93 | 1.43 | 0.83 |
| **2158** | 0.78 | 1.11 | 0.75 | 0.35 | 0.09 | 0.13 | 0.54 | 0.47 | 0.83 | 0.68 | 1.35 | 1.28 |
| **accD** | 1.05 | 1.01 | 1.16 | 0.39 | 0.13 | 0.12 | 0.87 | 0.51 | 0.98 | 1.04 | 1.17 | 1.49 |
| **trxB2** | 0.95 | 0.99 | 0.74 | 0.79 | 1.09 | 1.24 | 1.26 | 0.44 | 0.85 | 1.07 | 1.50 | 1.54 |
| **gltX** | 0.85 | 1.07 | 0.81 | 0.46 | 0.12 | 0.20 | 0.94 | 0.46 | 0.72 | 0.82 | 1.56 | 1.10 |
| **322** | 0.86 | 1.18 | 0.96 | 0.81 | 1.13 | 1.06 | 0.89 | 0.62 | 0.81 | 0.92 | 1.53 | 0.98 |
| **gyrB** | 0.89 | 1.04 | 0.64 | 0.82 | 0.88 | 1.29 | 0.91 | 0.78 | 0.83 | 1.32 | 1.65 | 1.28 |
| **sygB** | 0.95 | 1.09 | 0.75 | 0.55 | 1.32 | 1.44 | 1.15 | 0.68 | 1.28 | 1.25 | 1.33 | 1.18 |
| **pgsA** | 0.77 | 0.96 | 0.70 | 0.76 | 0.70 | 0.81 | 1.12 | 0.54 | 0.96 | 1.22 | 1.77 | 1.61 |
| **1746** | 0.79 | 1.00 | 0.82 | 0.74 | 1.10 | 1.28 | 1.37 | 0.64 | 0.90 | 0.93 | 1.52 | 1.18 |
| **rmlC** | 0.91 | 0.90 | 0.70 | 0.96 | 0.55 | 0.65 | 1.01 | 0.66 | 1.01 | 1.06 | 1.69 | 1.39 |
| **420** | 0.93 | 0.94 | 0.77 | 0.53 | 0.28 | 0.35 | 0.82 | 0.65 | 1.17 | 0.89 | 1.63 | 1.18 |
| **rs5** | 0.60 | 0.77 | 0.79 | 0.14 | 0.01 | 0.01 | 1.04 | 0.74 | 0.91 | 0.69 | 1.27 | 0.89 |
| **kthY** | 0.93 | 1.16 | 1.07 | 0.43 | 0.10 | 0.12 | 1.46 | 0.66 | 0.94 | 1.01 | 1.43 | 1.40 |
| **rgpE** | 0.87 | 0.95 | 0.91 | 0.63 | 0.28 | 0.45 | 0.77 | 0.41 | 0.92 | 1.37 | 1.82 | 1.41 |
| **852** | 0.79 | 1.35 | 1.09 | 0.71 | 0.98 | 1.40 | 0.79 | 0.78 | 0.69 | 1.13 | 1.64 | 1.53 |
| **parE** | 0.94 | 0.94 | 1.23 | 0.62 | 0.39 | 0.44 | 1.17 | 0.79 | 0.82 | 0.59 | 1.89 | 0.96 |
| **170** | 0.78 | 1.05 | 1.17 | 0.46 | 0.29 | 0.48 | 1.07 | 0.96 | 1.00 | 1.20 | 1.80 | 1.21 |
| **419** | 0.73 | 0.90 | 0.96 | 0.45 | 0.77 | 1.04 | 1.49 | 0.95 | 0.95 | 1.10 | 1.82 | 1.04 |
| **gapC** | 0.84 | 0.86 | 0.85 | 0.33 | 0.06 | 0.09 | 0.82 | 0.90 | 0.78 | 1.17 | 1.61 | 1.22 |
| **304** | 1.11 | 1.06 | 1.16 | 0.82 | 1.22 | 1.34 | 1.01 | 0.81 | 1.16 | 1.25 | 1.68 | 1.33 |
| **htrA** | 0.74 | 0.91 | 1.45 | 0.76 | 1.22 | 1.50 | 1.12 | 0.99 | 0.89 | 1.14 | 1.88 | 1.77 |
| **409** | 0.83 | 0.99 | 1.16 | 0.76 | 0.31 | 0.20 | 1.05 | 0.93 | 1.02 | 1.37 | 1.72 | 1.40 |
| **865** | 1.01 | 0.95 | 1.15 | 0.18 | 0.06 | 0.06 | 0.81 | 0.72 | 0.83 | 0.97 | 1.34 | 1.36 |
| **rexB** | 0.86 | 0.95 | 1.05 | 0.62 | 0.92 | 1.32 | 1.65 | 0.53 | 0.81 | 0.56 | 1.43 | 0.91 |
| **pmgY** | 1.03 | 0.85 | 0.93 | 0.49 | 0.15 | 0.20 | 1.00 | 1.31 | 1.10 | 1.54 | 1.75 | 1.31 |
| **1465** | 0.70 | 0.77 | 0.79 | 0.64 | 0.27 | 0.30 | 0.98 | 0.39 | 0.94 | 1.03 | 2.21 | 1.31 |
| **rl17** | 0.96 | 0.81 | 1.18 | 0.24 | 0.04 | 0.07 | 0.85 | 0.51 | 0.80 | 0.61 | 1.22 | 0.91 |
| **481** | 0.92 | 1.01 | 1.25 | 0.67 | 0.67 | 0.71 | 1.20 | 1.14 | 0.84 | 1.08 | 1.93 | 1.06 |
| **1742** | 0.64 | 0.73 | 0.87 | 0.23 | 0.07 | 0.09 | 0.92 | 1.00 | 0.84 | 0.64 | 1.08 | 0.71 |
| **589** | 0.92 | 1.12 | 1.29 | 0.81 | 1.12 | 1.37 | 0.95 | 0.83 | 0.87 | 1.20 | 2.08 | 1.11 |
| **943** | 0.85 | 1.01 | 1.32 | 0.79 | 0.02 | 0.05 | 0.80 | 1.22 | 0.83 | 1.32 | 1.89 | 1.35 |
| **24** | 0.98 | 0.93 | 1.22 | 0.77 | 0.87 | 0.97 | 1.35 | 0.87 | 0.91 | 1.24 | 1.58 | 0.93 |
| **bccP** | 0.64 | 0.82 | 1.62 | 0.18 | 0.06 | 0.09 | 0.93 | 0.78 | 0.62 | 1.15 | 2.05 | 1.74 |
| **dnaJ** | 0.79 | 0.99 | 1.44 | 0.72 | 1.11 | 1.36 | 1.26 | 1.00 | 1.24 | 1.32 | 1.85 | 1.54 |
| **353** | 0.70 | 0.91 | 1.08 | 0.75 | 0.94 | 1.12 | 0.95 | 0.76 | 0.76 | 0.95 | 1.56 | 1.03 |
| **966** | 0.70 | 1.00 | 1.17 | 0.61 | 0.33 | 0.39 | 1.12 | 0.69 | 1.17 | 1.01 | 1.65 | 1.18 |
| **nrdG** | 1.09 | 0.88 | 0.93 | 0.82 | 0.95 | 1.33 | 1.02 | 1.24 | 0.84 | 1.55 | 1.67 | 1.29 |
| **415** | 0.90 | 0.78 | 0.85 | 0.82 | 1.01 | 1.45 | 0.78 | 1.08 | 0.73 | 1.14 | 1.35 | 1.24 |
| **dnaX** | 0.92 | 1.07 | 1.49 | 0.69 | 1.14 | 1.49 | 0.74 | 0.40 | 0.84 | 0.65 | 1.78 | 1.25 |
| **kguA** | 0.88 | 0.74 | 0.68 | 0.67 | 0.63 | 0.89 | 0.69 | 0.97 | 0.84 | 1.22 | 2.06 | 0.85 |
| **368** | 0.76 | 1.11 | 1.10 | 0.36 | 1.08 | 1.42 | 1.05 | 0.92 | 0.85 | 0.78 | 1.34 | 0.98 |
| **nadE** | 1.04 | 1.02 | 0.99 | 0.68 | 1.13 | 1.31 | 0.93 | 0.87 | 0.89 | 1.17 | 1.68 | 1.10 |
| **proS** | 1.01 | 1.09 | 0.56 | 0.22 | 0.08 | 0.13 | 1.10 | 0.97 | 0.74 | 1.23 | 1.68 | 1.03 |
| **ftsZ** | 0.73 | 0.92 | 0.96 | 0.28 | 0.03 | 0.05 | 1.41 | 1.03 | 0.91 | 1.12 | 1.68 | 1.10 |
| **965** | 0.79 | 1.13 | 1.08 | 0.52 | 0.71 | 0.86 | 0.98 | 0.78 | 0.79 | 1.09 | 1.20 | 1.18 |
| **247** | 0.76 | 1.00 | 1.21 | 0.73 | 1.02 | 1.40 | 1.24 | 1.17 | 0.94 | 1.44 | 1.97 | 1.64 |
| **fabF** | 0.67 | 0.76 | 0.64 | 0.24 | 0.03 | 0.03 | 0.97 | 0.90 | 0.86 | 1.17 | 1.80 | 1.03 |
| **993** | 0.77 | 0.96 | 0.84 | 0.74 | 1.06 | 1.24 | 1.13 | 0.57 | 0.95 | 0.68 | 1.38 | 1.05 |
| **714** | 0.96 | 0.64 | 1.01 | 0.24 | 0.03 | 0.07 | 0.78 | 1.08 | 0.89 | 1.47 | 1.98 | 1.16 |
| **sygA** | 0.79 | 0.80 | 1.02 | 0.32 | 0.16 | 0.28 | 1.02 | 0.50 | 0.85 | 1.52 | 1.76 | 1.42 |
| **pth** | 0.83 | 0.90 | 1.41 | 0.92 | 1.16 | 1.35 | 0.87 | 0.38 | 0.71 | 1.19 | 2.01 | 1.58 |
| **169** | 0.86 | 0.93 | 1.40 | 0.25 | 0.16 | 0.27 | 1.18 | 1.13 | 0.71 | 1.50 | 2.23 | 1.42 |
| **thrC** | 0.68 | 0.82 | 0.89 | 0.64 | 0.52 | 0.87 | 0.95 | 1.44 | 0.82 | 1.39 | 1.86 | 1.16 |
| **pfk** | 0.68 | 1.17 | 1.22 | 0.27 | 0.07 | 0.09 | 1.07 | 1.41 | 0.86 | 1.49 | 2.15 | 1.18 |
| **rl24** | 0.79 | 0.91 | 1.10 | 0.23 | 0.05 | 0.07 | 1.07 | 1.34 | 0.75 | 1.59 | 1.82 | 1.09 |
| **940** | 0.86 | 0.87 | 0.77 | 0.60 | 1.24 | 1.36 | 1.25 | 0.84 | 1.00 | 1.75 | 2.19 | 1.10 |
| **sys** | 0.86 | 0.82 | 1.04 | 0.41 | 0.37 | 0.48 | 0.99 | 1.07 | 0.80 | 1.35 | 1.63 | 1.20 |
| **2143** | 0.79 | 0.93 | 1.05 | 0.63 | 0.69 | 1.08 | 0.91 | 1.09 | 0.76 | 1.47 | 2.01 | 1.62 |
| **pstB** | 0.74 | 0.93 | 0.89 | 0.38 | 0.14 | 0.22 | 0.89 | 0.68 | 0.97 | 1.14 | 1.84 | 1.25 |
| **capP** | 0.86 | 0.77 | 1.03 | 0.41 | 0.24 | 0.17 | 0.78 | 0.30 | 0.64 | 0.92 | 1.93 | 0.94 |
| **1820** | 0.92 | 0.78 | 0.98 | 0.56 | 0.16 | 0.21 | 0.87 | 1.07 | 0.84 | 1.47 | 1.96 | 1.18 |
| **nusA** | 0.87 | 0.83 | 1.15 | 0.77 | 0.74 | 1.05 | 0.86 | 0.80 | 0.69 | 1.10 | 1.86 | 1.25 |
| **dnaA** | 0.75 | 0.84 | 1.34 | 0.55 | 0.89 | 1.12 | 0.78 | 1.00 | 0.26 | 1.45 | 2.05 | 1.52 |
| **murF** | 0.82 | 0.80 | 1.15 | 0.17 | 0.07 | 0.09 | 0.92 | 0.97 | 0.67 | 1.56 | 2.03 | 1.17 |
| **393** | 0.88 | 0.90 | 1.23 | 0.54 | 0.99 | 1.27 | 0.81 | 0.88 | 0.81 | 1.48 | 2.18 | 1.47 |
| **715** | 0.83 | 1.01 | 1.00 | 0.34 | 0.30 | 0.42 | 0.98 | 1.11 | 0.85 | 1.22 | 2.10 | 1.49 |
| **vicR** | 0.95 | 1.31 | 0.92 | 0.65 | 1.04 | 1.30 | 1.15 | 1.23 | 0.79 | 1.45 | 2.33 | 1.53 |
| **glnA** | 0.86 | 0.93 | 1.55 | 0.59 | 1.25 | 1.56 | 1.11 | 1.05 | 1.29 | 1.54 | 2.02 | 1.52 |
| **650** | 0.72 | 0.95 | 1.25 | 0.19 | 0.08 | 0.08 | 0.78 | 1.06 | 0.68 | 1.19 | 2.16 | 1.18 |
| **2149** | 0.81 | 0.86 | 0.65 | 0.64 | 0.79 | 0.84 | 0.46 | 0.76 | 0.74 | 1.66 | 1.48 | 1.44 |
| **tkt** | 0.86 | 1.11 | 0.77 | 0.32 | 0.12 | 0.12 | 1.11 | 0.82 | 0.99 | 1.29 | 1.97 | 1.09 |
| **dnaG** | 0.72 | 0.90 | 0.61 | 0.40 | 0.19 | 0.19 | 1.12 | 0.58 | 0.81 | 1.29 | 1.97 | 1.01 |
| **2135** | 0.96 | 0.96 | 1.15 | 0.21 | 0.36 | 0.58 | 0.78 | 0.95 | 1.18 | 1.46 | 1.74 | 1.43 |
| **357** | 0.81 | 0.95 | 1.07 | 0.14 | 0.09 | 0.11 | 0.88 | 0.88 | 0.88 | 1.47 | 1.66 | 1.56 |
| **ftsH** | 0.75 | 0.92 | 1.09 | 0.30 | 0.26 | 0.44 | 0.73 | 2.00 | 0.92 | 1.21 | 1.35 | 1.61 |
| **pgi** | 0.90 | 0.97 | 0.99 | 0.44 | 0.15 | 0.20 | 1.12 | 0.68 | 1.17 | 1.39 | 1.94 | 1.27 |
| **cysS** | 0.72 | 0.89 | 1.11 | 0.52 | 0.66 | 0.97 | 1.25 | 0.54 | 0.88 | 1.34 | 1.78 | 1.46 |
| **824** | 0.91 | 1.03 | 1.25 | 0.51 | 0.21 | 0.30 | 0.66 | 0.88 | 0.86 | 1.15 | 1.83 | 1.19 |
| **priA** | 0.86 | 0.97 | 1.30 | 0.67 | 0.94 | 1.38 | 0.60 | 1.20 | 1.24 | 1.20 | 2.01 | 1.46 |
| **gbpB** | 0.84 | 0.88 | 1.28 | 0.25 | 0.10 | 0.16 | 1.33 | 0.98 | 0.97 | 1.24 | 1.89 | 1.31 |
| **697** | 0.82 | 0.94 | 1.22 | 0.71 | 1.15 | 1.33 | 1.19 | 0.68 | 1.04 | 0.95 | 1.49 | 1.40 |
| **2151** | 0.85 | 0.84 | 0.82 | 0.74 | 1.20 | 1.10 | 1.34 | 0.90 | 0.95 | 1.41 | 1.91 | 1.31 |
| **thyA** | 0.86 | 1.03 | 0.72 | 0.50 | 0.94 | 0.90 | 1.02 | 0.68 | 1.16 | 1.36 | 1.79 | 1.25 |
| **secA** | 0.77 | 1.05 | 0.87 | 0.47 | 1.22 | 1.48 | 1.27 | 0.51 | 1.62 | 1.61 | 2.02 | 1.51 |
| **rpoZ** | 0.79 | 1.07 | 1.02 | 1.04 | 0.21 | 0.10 | 0.87 | 1.41 | 1.16 | 1.13 | 0.98 | 0.81 |
| **adk** | 0.88 | 1.00 | 0.89 | 0.93 | 0.11 | 0.06 | 1.36 | 1.63 | 0.86 | 1.17 | 1.33 | 0.80 |
| **murB** | 0.98 | 1.05 | 1.11 | 0.44 | 0.19 | 0.22 | 1.00 | 1.40 | 1.22 | 1.16 | 1.42 | 0.92 |
| **1821** | 0.99 | 1.02 | 1.26 | 0.70 | 0.15 | 0.14 | 1.14 | 1.26 | 1.48 | 1.27 | 1.40 | 1.05 |
| **ftsL** | 0.89 | 1.09 | 1.27 | 0.27 | 0.05 | 0.03 | 1.13 | 1.04 | 1.63 | 0.87 | 1.22 | 0.85 |
| **rgpG** | 0.98 | 1.00 | 1.09 | 1.85 | 0.96 | 0.16 | 1.13 | 0.70 | 1.11 | 1.15 | 0.93 | 0.67 |
| **rrf1** | 1.02 | 0.90 | 1.06 | 1.64 | 0.95 | 0.90 | 1.07 | 0.88 | 0.83 | 1.32 | 1.48 | 0.84 |
| **secY** | 0.96 | 0.97 | 1.19 | 0.61 | 0.09 | 0.06 | 1.28 | 1.04 | 1.03 | 1.11 | 1.22 | 0.76 |
| **gyrA** | 0.93 | 1.15 | 1.04 | 1.52 | 1.08 | 0.84 | 0.61 | 0.90 | 1.33 | 1.19 | 1.05 | 0.49 |
| **352** | 0.83 | 1.15 | 1.04 | 0.95 | 0.67 | 0.69 | 0.38 | 1.03 | 1.12 | 0.96 | 1.11 | 0.57 |
| **901** | 0.97 | 1.03 | 1.08 | 0.91 | 0.21 | 0.22 | 1.00 | 1.26 | 0.98 | 0.96 | 1.05 | 0.65 |
| **akh** | 1.00 | 1.04 | 1.07 | 1.37 | 0.42 | 0.34 | 0.83 | 1.08 | 0.73 | 0.96 | 1.22 | 0.84 |
| **argS** | 0.90 | 1.05 | 1.18 | 0.19 | 0.04 | 0.03 | 1.07 | 1.37 | 1.37 | 1.29 | 1.49 | 0.83 |
| **1588** | 1.08 | 1.14 | 1.39 | 0.82 | 0.50 | 0.58 | 0.78 | 1.44 | 1.01 | 1.39 | 1.63 | 1.01 |
| **dnaN** | 0.89 | 0.97 | 1.14 | 1.65 | 1.16 | 1.27 | 0.87 | 1.16 | 1.06 | 0.90 | 1.30 | 0.84 |
| **rgpB** | 0.88 | 1.11 | 1.43 | 0.71 | 0.20 | 0.15 | 1.10 | 0.99 | 1.25 | 1.10 | 1.49 | 0.78 |
| **r15** | 0.90 | 0.84 | 1.20 | 0.30 | 0.02 | 0.01 | 0.87 | 0.74 | 1.02 | 1.30 | 1.34 | 0.68 |
| **rgpA** | 1.10 | 1.05 | 1.15 | 0.75 | 0.17 | 0.15 | 0.95 | 1.04 | 1.27 | 1.16 | 1.43 | 0.62 |
| **pbp2x** | 0.92 | 0.99 | 1.23 | 0.29 | 0.01 | -0.01 | 0.80 | 1.22 | 1.02 | 1.00 | 1.08 | 0.42 |
| **675** | 0.83 | 0.97 | 1.21 | 0.82 | 0.30 | 0.33 | 0.75 | 1.13 | 1.10 | 1.20 | 1.36 | 0.68 |
| **937** | 0.65 | 1.10 | 1.21 | 0.61 | 0.01 | 0.01 | 1.25 | 0.97 | 0.83 | 1.10 | 1.29 | 0.88 |
| **groEL** | 1.05 | 1.01 | 1.26 | 1.90 | 0.47 | 0.45 | 0.89 | 0.84 | 0.72 | 1.19 | 1.46 | 0.86 |
| **murC** | 0.79 | 1.02 | 1.32 | 0.33 | 0.02 | 0.03 | 1.14 | 1.28 | 0.83 | 1.21 | 1.70 | 1.04 |
| **yidC2** | 1.06 | 1.13 | 1.31 | 1.34 | 0.79 | 0.91 | 1.33 | 1.06 | 0.88 | 1.09 | 1.66 | 1.10 |
| **421** | 0.97 | 0.95 | 1.41 | 0.79 | 0.24 | 0.29 | 1.28 | 1.12 | 1.08 | 1.18 | 1.36 | 1.07 |
| **rgpD** | 0.88 | 0.90 | 1.39 | 0.50 | 0.87 | 0.75 | 0.99 | 1.11 | 1.04 | 1.28 | 1.41 | 1.36 |
| **fabG** | 0.83 | 0.75 | 1.00 | 0.30 | 0.04 | 0.03 | 1.00 | 1.41 | 0.90 | 0.99 | 0.97 | 0.61 |
| **rpoD** | 0.73 | 0.98 | 1.22 | 0.23 | 0.03 | 0.01 | 1.10 | 1.20 | 1.22 | 1.15 | 1.21 | 0.86 |
| **ftsA** | 0.80 | 1.03 | 1.35 | 1.09 | 0.76 | 0.72 | 0.92 | 1.21 | 1.11 | 1.31 | 1.30 | 0.95 |
| **696** | 0.84 | 1.17 | 1.65 | 1.35 | 0.82 | 0.78 | 0.47 | 0.88 | 0.79 | 0.98 | 1.32 | 0.71 |
| **rl1** | 0.73 | 1.05 | 1.20 | 0.18 | 0.01 | 0.01 | 1.18 | 0.96 | 1.07 | 0.94 | 1.28 | 0.92 |
| **938** | 0.86 | 0.95 | 1.39 | 0.51 | 0.00 | 0.00 | 0.91 | 1.14 | 0.89 | 0.98 | 1.28 | 1.36 |
| **ilvE** | 0.90 | 1.07 | 0.97 | 1.71 | 0.92 | 1.03 | 1.14 | 1.08 | 0.92 | 1.11 | 1.71 | 1.23 |
| **rpoC** | 0.99 | 1.06 | 1.42 | 0.52 | 0.02 | 0.05 | 1.43 | 0.95 | 0.88 | 1.13 | 1.60 | 1.03 |
| **plsX** | 1.04 | 1.01 | 1.63 | 0.77 | 0.36 | 0.44 | 1.40 | 0.97 | 0.85 | 1.27 | 1.52 | 1.18 |
| **pyrG** | 0.84 | 0.90 | 1.48 | 0.61 | 0.07 | 0.09 | 1.02 | 0.87 | 0.88 | 1.04 | 1.46 | 1.06 |
| **rs17** | 0.08 | 0.03 | 0.05 | -0.05 | -0.02 | -0.03 | 0.10 | 0.11 | 0.10 | 0.06 | 0.08 | 0.05 |
| **517** | 0.80 | 1.29 | 1.49 | 1.27 | 0.49 | 0.47 | 1.19 | 1.15 | 0.87 | 1.16 | 1.29 | 0.97 |
| **ldh** | 0.89 | 1.08 | 1.34 | 1.81 | 1.11 | 1.15 | 1.39 | 1.50 | 1.28 | 1.03 | 1.46 | 0.92 |
| **groES** | 0.77 | 1.09 | 1.58 | 1.57 | 0.84 | 0.91 | 1.06 | 1.11 | 1.08 | 1.26 | 1.64 | 1.26 |
| **pfs** | 0.80 | 1.07 | 1.38 | 1.44 | 1.09 | 1.21 | 0.86 | 0.86 | 0.84 | 1.22 | 1.69 | 1.20 |
| **958** | 0.65 | 1.21 | 1.25 | 0.09 | 0.03 | 0.03 | 1.01 | 1.20 | 1.03 | 1.13 | 1.46 | 1.36 |
| **1428** | 0.98 | 0.95 | 1.44 | 1.74 | 0.71 | 0.51 | 1.39 | 1.03 | 1.07 | 0.77 | 1.59 | 0.96 |
| **rmlA** | 0.99 | 1.08 | 1.52 | 1.28 | 0.01 | 0.02 | 0.94 | 1.01 | 1.00 | 1.17 | 1.71 | 1.41 |
| **fbaA** | 0.99 | 0.97 | 1.45 | 0.56 | 0.03 | 0.06 | 1.47 | 0.91 | 1.07 | 1.03 | 1.71 | 1.00 |
| **rnpB** | 0.87 | 0.97 | 1.61 | 0.87 | 0.24 | 0.30 | 1.37 | 0.91 | 0.76 | 1.24 | 1.62 | 1.41 |
| **syl** | 0.81 | 1.01 | 1.60 | 0.54 | 0.22 | 0.34 | 1.27 | 1.16 | 0.97 | 1.13 | 1.51 | 1.05 |
| **if1** | 0.90 | 1.07 | 1.46 | 0.16 | 0.02 | 0.02 | 0.97 | 1.00 | 0.99 | 0.87 | 1.26 | 1.07 |
| **1051** | 0.86 | 0.96 | 1.79 | 1.12 | 0.60 | 0.56 | 1.23 | 1.46 | 0.93 | 1.15 | 1.68 | 1.47 |
| **ftsX** | 1.05 | 1.10 | 1.65 | 1.72 | 0.99 | 1.19 | 1.30 | 1.28 | 1.15 | 1.04 | 1.40 | 1.57 |
| **358** | 0.95 | 0.97 | 1.50 | 0.17 | 0.01 | 0.05 | 1.25 | 1.01 | 0.95 | 0.87 | 1.58 | 1.67 |
| **prs** | 0.83 | 1.08 | 1.72 | 0.47 | 0.18 | 0.19 | 1.14 | 0.97 | 0.96 | 1.18 | 1.52 | 1.41 |
| **rpoB** | 1.05 | 1.19 | 1.52 | 0.56 | 0.05 | 0.04 | 0.98 | 1.13 | 0.86 | 1.08 | 1.35 | 1.39 |
| **syfA** | 0.86 | 1.01 | 1.51 | 0.44 | 0.05 | 0.06 | 1.30 | 1.16 | 0.89 | 1.15 | 1.69 | 1.52 |
| **624** | 0.93 | 1.14 | 1.78 | 0.92 | 0.31 | 0.47 | 1.87 | 1.21 | 0.92 | 1.07 | 1.77 | 1.47 |
| **dnaC** | 0.92 | 0.93 | 1.46 | 1.18 | 0.22 | 0.21 | 1.35 | 1.18 | 0.77 | 1.42 | 1.91 | 1.88 |
| **773** | 0.64 | 0.79 | 1.37 | 0.35 | 0.05 | 0.08 | 1.13 | 0.85 | 0.80 | 1.16 | 1.86 | 1.40 |
| **1134** | 0.83 | 0.98 | 1.51 | 0.78 | 0.18 | 0.28 | 1.07 | 0.97 | 0.75 | 1.08 | 1.89 | 1.59 |
| **dnaI** | 0.89 | 1.06 | 1.75 | 1.55 | 0.76 | 0.92 | 1.49 | 1.02 | 0.99 | 1.30 | 2.12 | 1.62 |
| **divIC** | 0.79 | 1.12 | 1.69 | 0.70 | 0.05 | 0.07 | 1.29 | 1.20 | 1.03 | 1.17 | 1.88 | 1.36 |
| **1211** | 0.90 | 1.06 | 1.60 | 1.46 | 0.93 | 1.05 | 1.18 | 1.08 | 0.86 | 1.28 | 2.09 | 0.80 |
| **dfrA** | 0.70 | 1.01 | 1.01 | 0.92 | 0.89 | 0.76 | 1.19 | 1.20 | 0.96 | 1.23 | 1.95 | 0.96 |
| **323** | 0.91 | 0.95 | 1.41 | 0.44 | 0.05 | 0.05 | 1.53 | 0.89 | 0.96 | 1.05 | 1.76 | 0.76 |
| **syfB** | 1.03 | 1.15 | 1.54 | 0.52 | 0.15 | 0.17 | 1.27 | 1.22 | 0.85 | 1.11 | 1.91 | 1.09 |
| **359** | 0.81 | 0.99 | 1.58 | 1.25 | 0.76 | 1.01 | 1.43 | 1.05 | 1.00 | 1.08 | 1.96 | 1.34 |
| **murC2** | 0.92 | 0.92 | 1.46 | 0.62 | 0.03 | 0.02 | 1.32 | 1.21 | 0.93 | 1.10 | 1.80 | 1.37 |
| **murI** | 0.89 | 0.95 | 1.24 | 0.59 | 0.11 | 0.21 | 1.35 | 1.13 | 0.80 | 1.19 | 2.11 | 1.40 |
| **ffh** | 0.93 | 0.94 | 1.27 | 1.49 | 0.76 | 1.13 | 1.45 | 1.01 | 0.83 | 1.21 | 1.70 | 1.44 |
| **grpE** | 1.02 | 0.94 | 1.42 | 1.76 | 1.00 | 1.23 | 1.24 | 1.13 | 0.72 | 1.32 | 1.34 | 1.02 |
| **topA** | 0.90 | 1.01 | 1.50 | 1.07 | 0.78 | 0.84 | 1.44 | 1.00 | 0.84 | 1.14 | 1.49 | 1.06 |
| **1801** | 1.12 | 1.23 | 1.45 | 1.10 | 0.31 | 0.32 | 1.20 | 0.88 | 0.85 | 1.17 | 1.89 | 1.32 |
| **849** | 0.66 | 1.01 | 1.07 | 0.43 | 0.21 | 0.18 | 1.11 | 0.99 | 0.94 | 1.16 | 1.73 | 0.75 |
| **385** | 0.89 | 0.96 | 0.80 | 1.60 | 0.18 | 0.12 | 0.69 | 0.82 | 0.94 | 0.99 | 1.63 | 1.28 |
| **pstC** | 0.80 | 1.19 | 1.01 | 0.31 | 0.07 | 0.07 | 1.15 | 1.07 | 0.89 | 0.91 | 1.32 | 0.71 |
| **dfp** | 0.91 | 1.03 | 1.42 | 1.16 | 0.73 | 0.72 | 1.11 | 1.17 | 1.11 | 1.11 | 1.76 | 0.98 |
| **668** | 1.01 | 1.09 | 1.51 | 0.95 | 0.78 | 1.04 | 1.13 | 1.20 | 0.88 | 1.16 | 1.77 | 1.44 |
| **murM** | 0.81 | 1.20 | 1.25 | 0.32 | 0.04 | 0.06 | 1.19 | 1.21 | 1.11 | 1.27 | 1.99 | 1.59 |
| **369** | 0.82 | 0.94 | 1.59 | 1.91 | 0.96 | 1.37 | 1.18 | 0.92 | 1.18 | 1.33 | 2.22 | 1.07 |
| **pgk** | 1.13 | 0.98 | 1.44 | 1.58 | 1.27 | 1.32 | 1.23 | 1.29 | 1.12 | 1.39 | 2.16 | 1.23 |
| **atpB** | 0.89 | 0.91 | 1.24 | 0.57 | 0.86 | 0.99 | 1.01 | 1.08 | 1.12 | 1.25 | 2.00 | 0.88 |
| **mvaA** | 0.93 | 0.98 | 1.47 | 1.90 | 1.22 | 1.37 | 1.36 | 1.09 | 0.91 | 1.25 | 1.60 | 1.45 |
